# Psychiatric morbidity in outpatients of gynecological oncology clinic in a tertiary care hospital

**DOI:** 10.4103/0019-5545.74307

**Published:** 2010

**Authors:** Rohan Dilip Mendonsa, Prakash Appaya

**Affiliations:** Department of Psychiatry, Yenepoya Medical College, Mangalore, Karnataka, India; 1 St John’s Medical College, Bangalore, Karnataka, India

**Keywords:** Anxiety, depression, gynecological cancer

## Abstract

**Background:**

Psychiatric morbidity in gynecological oncology patients is relatively less studied.

**Aims:**

This cross-sectional observational study was undertaken to assess the common psychiatric disorders in women who consult the gynecological oncology outpatients’ department.

**Materials and methods:**

We assessed a total of 101 outpatients who were recruited by convenience method of sampling. The main outcome measures were PRIME-MD PHQ diagnoses, gynecological and sociodemographic profiles.

**Results:**

Psychiatric disorders as detected by PRIME –MD PHQ were diagnosed in 44% of the patients. Mood disorders were most common. Major depression was present in 25.7% of patients. Anxiety disorders were diagnosed in 16.8% of the patients. Among 44 patients with a psychiatric diagnosis only one patient was on psychiatric treatment. Major depression was much more common (34.4%) in cancer patients than in women with benign conditions (16.6%).

**Conclusion:**

The findings of our study reveal a high rate of psychiatric morbidity in the gynecological oncology outpatients.

## INTRODUCTION

Several studies have indicated an overlap of gynecological and psychiatric problems.[1–4] However, gynecological cancer has received much less attention than breast cancer in terms of studying the psychiatric co-morbidity. Emotional reactions of newly diagnosed gynecologic cancer patients are severe. Reports of clinically significant depressive and anxiety symptoms are common.[5,6] At least 20% of patients with gynecological cancers are so distressed that they merit a psychiatric diagnosis, such as major depressive disorder.[7] In contrast, the incidence of major depression in the general population is 5–6%.[8]

Genital neoplasia affects a woman’s life by threatening her self-concept, body image, sense of feminity and sexuality.[9] The emotional reactions could be attributed to the devastation of a cancer diagnosis, but at least one study shows that individuals are significantly distressed long before a diagnosis of malignancy is learned.[10] Fowler *et al*. found that unlike many newly diagnosed cancer patients who report they have felt “normal” in the preceding months, women referred to a gynecological oncologist have not. Depression, anxiety, and adjustment disorders have been found to occur with heightened frequency in patients with gynecological cancers, and appear to worsen over the course of treatment persisting well after the initial diagnosis and therapy.[11,12] A study that compared benign breast biopsy patients, breast cancer mastectomy patients, and gynecological cancer patients found that severity of depression of the gynecological cancer patients was significantly higher than the other two groups.[13] A study that assessed the efficacy of psychological interventions in gynecological cancer patients suggests that individual or group therapy may be effective for this patient population.[14] Another study illustrated the efficacy of imipramine in depressed patients with gynecological cancers and concluded that treatment of depression is associated with better life adaptation.[15]

The literature suggests that patients with both benign and malignant gynecological conditions are at high risk of developing psychiatric disorders. Studies done in developed countries with good healthcare infrastructure report that majority of the patients with psychiatric morbidity remain undiagnosed and do not receive psychiatric treatment. Undiagnosed and untreated psychiatric morbidity might affect the treatment compliance and cause overall poor quality of life. Many depressed patients adhere poorly to treatment schedules and other recommendations; some may have reduced chance of survival. The magnitude of this problem could be much higher in a developing country like India. Hence, psychiatric morbidity in cancers of the female reproductive system may be particularly important to study in the Indian scenario. The findings will have important implications in women’s healthcare.

### Aims and objectives

1. To assess the frequency and nature of psychiatric morbidity among women attending the gynecological oncology outpatient clinic.
2. To compare the frequency and nature of psychiatric morbidity among gynecological outpatients with benign and malignant gynecological diseases.
3. To study the relationship between socio-demographic, psychosocial and clinical variables and the psychiatric morbidity in women attending the gynecological oncology clinic.

## MATERIALS AND METHODS

This clinical investigation was conducted in the gynecological oncology outpatient clinic of St. John’s Medical College Hospital, Bangalore over a period of one year from August 2005 to July 2006. This gynecological oncology clinic caters to both referred and walk-in patients. The treating team consists of a senior gynecological oncologist and several other gynecologists. The population from which the subjects for this investigation were drawn, comprised all women outpatients who attended the gynecological oncology clinic during the above-mentioned period regardless of their diagnosis. All women who attended the outpatient oncology clinic during this period on Mondays and Wednesdays were approached by one of the investigators requesting for participation in this study. All women outpatients above the age of 18 years who consented to take part in the study were included in the sample. Patients who could not communicate in any of the three languages namely, English, Hindi or Kannada were excluded. A total of 150 patients were approached of which 26 declined to participate in the study and 23 were excluded because of language difficulty. The sample consisted of 101 outpatients of the gynecological oncology clinic recruited for the investigation on a convenient non-random basis over a period of one year. Informed consent was obtained from all the subjects. After the gynecological consultation they were interviewed using a specially designed questionnaire to collect information regarding socio-demographic, medical and gynecological data. The medical and gynecological information were also gathered from the gynecologists as well as from the clinical case sheets. The PRIME MD-PHQ was administered to each subject and the responses were recorded. The investigator verified the patients’ responses and the criteria in the PRIME MD-PHQ were applied to make the diagnosis. The level of socio-occupational dysfunction due to symptoms mentioned in the questionnaire was recorded.

All statistical analyses were conducted by using Statistical Package for the Social Sciences (SPSS, Inc, Chicago, IL). Primary analyses tested the association of socio-demographic and gynecology symptom variables with psychiatric morbidity. Next, subjects were divided into three groups based on their gynecological status. The first group consisted of women who were undergoing evaluation. Some of the patients of this group might have been diagnosed to have cancer at a later date. Hence this group was presumed to have a mixed population in terms of cancer versus benign conditions. This group was called ‘under evaluation group’. They were 42 in number. The second group consisted of women who had a benign gynecological diagnosis. This was called the ‘benign group’. They were 26 in number. The third group was made up of women who had gynecological cancer. This was called the ‘cancer group’. This group had 33 subjects. The three gynecological status groups were compared in terms of the associated psychiatric morbidity and other psychosocial variables. Then the benign group and the cancer group were compared for statistically significant differences in terms of psychiatric morbidity and other psychosocial variables.

The PRIME-MD PHQ was developed by Spitzer and colleagues.[3] In addition to assessing mood, anxiety, eating, alcohol and somatoform disorders, as in the original PRIME-MD, the PRIME-MD PHQ screens for disorders that are especially common among women, such as Pre-Menstrual Syndrome (PMS), post-partum and menopausal mood disorders and post-traumatic stress disorders. The PRIME-MD PHQ also includes questions about menstruation, pregnancy and childbirth and enquires about ten common psychosocial stressors that have occurred during the last month.

## RESULTS

### Socio-demographic characteristics

The mean age of the subjects was 45.8 years. The other characteristics of the sample are given in Table 1.

**Table 1:** Socio-demographic profile and psychiatric morbidity

Variables	Psy positive (N=44) %	Psy negative (N=57) %	Chisquare value	*P* value	df
Religion					
Hindu	50.6	49.4		
Muslim	0	100	9.731	0.021	3
Others	14.3	85.7			
Marital status					
Single	40	60			
Married	42.5	57.5	2.24	0.523	3
Separated	20	80			
Widow	55.6	44.4			
Educational status					
Illiterate	55.2	44.8			
Literate	44.9	55.1	4.482	0.106	2
College	26	74			
Family type					
Nuclear	45.8	54.2			
Ext/joint	44.8	55.2	1.000	0.606	2
Others	30.8	69.2			
Economic status					
Lower	49	51			
Middle	39	61	2.562	0.278	2
Higher	0	100			
Locality					
Urban	40.9	59.1	0.546	0.460	1
Occupation					
Housewives	45.7	54.3	0.429	0.513	1
Working	38.7	61.3			

### Gynecological/medical profile

Menstruation-related symptoms (31%), abdominal pain (24%) and postmenopausal bleed were the most common gynecological symptoms. About 2/5^th^of the subjects had gynecological symptoms lasting more than six months. Fourteen percent of subjects had undergone hysterectomy. Forty-two of them were under evaluation and their cancer/non-cancer status was not yet confirmed. Thirty-three of them had a diagnosis of gynecological cancer, of which cervical cancer was the most common. Twenty-six (26) women had benign gynecological conditions, of which fibroid and benign ovarian tumors were most common. Among the 33 cancer patients, 29 were recently diagnosed (less than six months back). Five of the cancer patients did not know about their diagnosis. Six of the cancer patients were on chemotherapy and two of them were receiving radiotherapy at the time of their participation in the study. Hypertension (14%) and diabetes mellitus (12%) were the most common co- morbid medical conditions.

### Psychiatric morbidity

**Figure 1:**
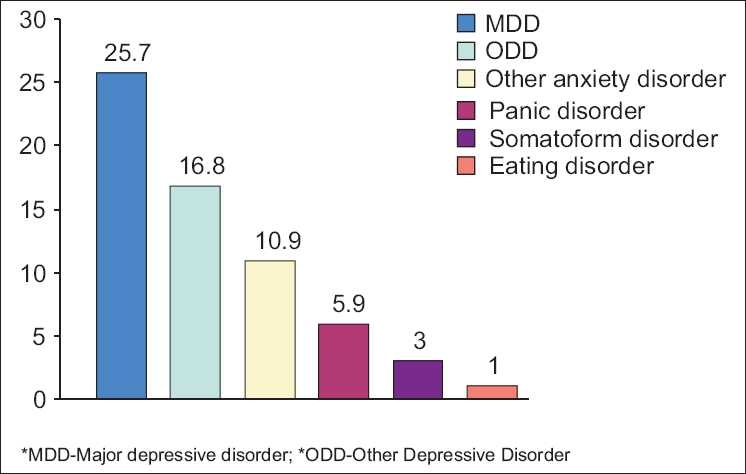
Prevalence of psychiatric disorders in the sample (in percentages)

Forty-four percent (44%) of women, who participated in the study had at least one PRIME-MD PHQ diagnosis. Threshold diagnoses like major depressive disorder (MDD), panic disorder, and other anxiety disorders were present in 37% of the women; 7% had sub-threshold diagnoses only (e.g., other depressive disorder). MDD was detected in 25.7%. This was followed by other depressive disorder (16.8%), other anxiety disorder (10.9%), panic disorder (5.9%), somatoform disorder (3%), and eating disorder (1%) [Figure 1]. In addition, 6% of the women had symptoms suggestive of pre-menstrual dysphoric disorder and 1% had features suggestive of post-traumatic stress disorder. Depression and anxiety was co-morbid in 8% of the subjects. Past history of psychiatric illness was reported by 9% of the subjects. Most of them had depressive disorders.

### Psychosocial stress

A composite stress score was computed based on the responses of the participants to a set of 10 questions in the PRIME-MD PHQ, addressing the most common stresses found in women. Mean stress score was 2.99. About 72% of the subjects had stress score of 2 or more i.e. they reported at least one psychosocial stressor about which they were ‘bothered a lot’.

### Socio-demographic profile and psychiatric morbidity

Higher rates of psychiatric morbidity were found in widows than those who were living with their partners (55.6% vs. 42.5%); in those who were illiterate than literate (55.2% vs. 44.9%); in those from nuclear families (45.8% vs. 44.8%); in those from lower socio-economic status than middle (49% vs. 39%); in those from rural than urban areas (49% vs. 40.9%); and in housewives than in working women (45.7% than 38.7%). But the analysis done to compare socio-demographic variables between women with psychiatric morbidity and women without psychiatric morbidity using chi-square test showed no difference of statistical significance [Table 1]. Women with psychiatric morbidity did have significantly higher psychosocial stressors. The mean stress score in the psychiatric morbidity group was 3.42, whereas it was 2.72 in the ‘no psychiatric morbidity’ group. This was statistically significant (*P*=0.028). Fifty-seven percent (57%) of those with psychiatric morbidity reported socio-occupational dysfunction, 20% reported mild dysfunction (Reported ’somewhat difficult’ to do the daily chores), 14% reported moderate dysfunction (Very difficult) and 23% reported severe dysfunction (’Extremely difficult’).

### Gynecological profile and psychiatric morbidity

Psychiatric morbidity was present in 35.2% of the postmenopausal women as compared to 48.4% of premenopausal women. About 36% of the women in the ’under evaluation group’ had psychiatric morbidity as compared to 46% in benign group, and 50% in cancer group. When gynecological symptoms were compared for the presence of psychiatric morbidity, postmenopausal bleeding was associated with the highest psychiatric morbidity (60%). About 50% of those complaining of pain abdomen, and 48.4% of those having menstruation-related symptoms had psychiatric morbidity. Among the hysterectomized women, 41.4% had psychiatric morbidity compared to 57.2% of women who had not undergone hysterectomy. Women with gynecological symptoms for more than six months showed 63.5% incidence of psychiatric morbidity whereas only 30% of those with symptoms for less than six months had psychiatric morbidity. The Chi-square test showed that there was a statistically significant association between the duration of gynecological symptoms and psychiatric morbidity. But the women with psychiatric morbidity and those without it did not differ significantly in terms of their menopausal status, the presenting gynecological symptoms, the gynecological diagnosis and history of hysterectomy.

### Comparison of psychiatric morbidity across gynecological status-based groups

Fifty percent of the cancer group had psychiatric morbidity as compared to 42% of the benign group and 35.7% of the ‘under evaluation group’. Depressive disorders were found in 42.3% of women with benign conditions, 42.4% of the cancer group and 35.7% of the ‘under evaluation’ group. Major depressive disorder was remarkably more common in the cancer group (33.3%) as compared to the benign group (15.4%). However, there was no statistically significant difference between the two groups in terms of the psychiatric morbidity. The mean psychosocial stress score was higher in the cancer group (3.19) than the benign group (2.96). The statistical analysis however did not show any significant difference between the groups. Other depressive disorders were more common in the benign group (26.9%) compared to the cancer group (12%) and ’under evaluation’ group (11.9%). There was no significant difference between the groups in terms of the anxiety disorders. The incidence varied from 11.5% to 15% between the groups. Panic disorder was present in 6% of the cancer group subjects, 4.8% of the ’under evaluation’ group and 3.8% of the benign group subjects. Other anxiety disorder was highest in the benign group (15.4%) as compared to the ’under evaluation’ (9.5%) and cancer group (9%). Somatoform disorder was present in 7.8% of the subjects in the benign group, as compared to 4.8% in the ’under evaluation’ and none in the cancer group subjects. The only case of eating disorder (Bulimia Nervosa) detected was in the benign group. None of the subjects had alcohol abuse/dependence [Table 2].

**Table 2:** Distribution of PRIME-MD PHQ psychiatric diagnoses among the three gynecological diagnosis groups

PRIME-MD PHQ diagnoses	Under evaluation group N=4		Benign group (B) N=26		Gyn can group (G) N=33		Comparison between B and G	
	Num	%	Num	%	Num	%	Chi-square value	*P* value (d.f=1)
Any psychiatric diagnosis	15	35.7	11	42.3	16	50	0.095	0.757
Any mood disorder	15	35.7	11	42.3	14	42.4	0.024	0.877
Major depressive disorder	10	23.8	4	15.4	11	33.3	2.193	0.139
Other depressive disorder	5	11.9	7	26.9	4	12	2.413	0.120
Any anxiety disorder	5	11.9	3	11.5	5	15	1.09	0.741
Panic disorder	2	4.8	1	3.8	2	6	1.17	0.732
Other anxiety disorder	4	9.5	4	15.4	3	9	0.667	0.414
Somatoform disorder	2	4.8	2	7.8	0	0	1.35	0.244

## DISCUSSION

Though many studies have looked into the psychiatric morbidity in gynecological outpatients, the psychiatric disorders in gynecological oncology patients have been relatively less studied. No Indian study that we know of has assessed the psychiatric morbidity in gynecological oncology outpatients. The study revealed a significant psychiatric morbidity in the studied population. About 44% of the women showed at least one PRIME-MD PHQ diagnosis. About 7% had a subthreshold diagnosis only and the remaining 37% had threshold diagnosis. Depressive disorders and anxiety disorders were diagnosed in 42.5% and 16.8% respectively. The findings of the current study were comparable to another study which found clinically significant depressive and anxiety symptoms in 42% and 30% respectively.[10]

The most common psychiatric disorder in the subjects in our study was MDD (25.7%), a finding consistent with other studies done in gynecological outpatients. A significant number of our subjects had other depressive disorder (16.8%), other anxiety disorder (10.9%), pre-menstrual dysphoric disorder (6%), and panic disorder (5.9%). The women with psychiatric morbidity and those without psychiatric morbidity did not differ significantly on the socio-demographic variables. One of the reasons for this might be the sample size of the study, which was too small to permit testing of the interaction of the multiple significant variables.

Psychosocial stressors of the subjects of the study were quite high. Most of the women (72%) reported that they were ‘bothered a lot’ about at least one psychosocial stressor. The most common stressors were health-related (63%), financial (26%) and interpersonal in the family context (23%). About half the participants had socio-occupational dysfunction. The level of psychosocial stressors as calculated by the composite stress score was significantly higher in women with psychiatric morbidity than those without psychiatric morbidity (*P*<0.028). There was a preponderance of pre-menopausal women, and the variation in psychiatric morbidity with menopausal status and chronological age observed in the general population studies was not evident in the clinic sample. In fact, pre-menopausal women had higher psychiatric morbidity than post-menopausal women (48.4 vs. 35.2%). Also, past history of hysterectomy did not seem to contribute to psychiatric morbidity.

When different gynecological status groups were compared the psychiatric morbidity was highest in the cancer group (50%), followed by benign (46%) and ’under evaluation’ group (36%). Depressive disorders were the leading psychiatric morbidity in all the three groups. Anxiety disorders were also found across the groups. The depression seen in women with gynecological cancer tended to be severe as compared to women with benign gynecological conditions who were likely to have mild depressive symptoms. The two groups however did not differ significantly on the psychiatric psychosocial variables. This finding was consistent with the conclusion of a similar study which compared depressive and anxiety symptoms in women who had been referred for gynecological oncologist’s evaluation.[10]

A cross-sectional study has found that those with ovarian cancer or other, poorly differentiated tumors and/or those receiving triple-agent chemotherapy appear to be at increased risk of developing depression.[16] But our study did not look into different gynecological cancers nor staging of the cancer and its histopathology were considered while estimating the associated psychiatric morbidity. A longitudinal study that compared women with gynecological cancer with women with benign gynecological diagnosis has found significant anxiety scores in both the benign gynecology group and the cancer group but a depressed mood in only the cancer group (*P*<0.01).[6] In contrast our study found both depressive and anxiety symptoms in both the groups.

Studies in the past have reported that gynecological patients with menstrual problems and chronic abdominal pain are more likely to have psychiatric morbidity.[17] But, we found that women with different gynecological symptoms did not vary significantly in terms of the associated psychiatric morbidity. The only gynecological factor significantly associated with a high risk of psychiatric morbidity was the duration of gynecological symptoms. In women who were symptomatic for more than six months, psychiatric morbidity was present in 64%, whereas in those who were symptomatic for less than six months, it was present in only 30%. This was statistically significant (*P*<0.001).

What was more striking in this study was the fact that the great majority of cases had been unrecognized and untreated. Only one out of 44 women with PRIME-MD PHQ diagnosis was on psychiatric treatment. Diagnosing psychiatric morbidity in women in a gynecological oncology setting has many implications. Undiagnosed and untreated psychiatric morbidity might affect the treatment compliance and cause overall poor quality of life. In this study the psychiatric morbidity was associated with significant socio-occupational dysfunction. Mild, moderate, and severe dysfunction was found in 20%, 14%, and 23% respectively, among women with psychiatric morbidity. This emphasizes the importance of recognizing and treating the psychiatric disorders in this particular population. Another issue which is of importance, is proper training of clinicians to recognize and refer patients with common mental disorders. A large number of women experiencing psychological distress do not seek help from a mental health professional. An obstetrician–gynecologist may be these women’s only point of contact with a medical professional. In this regard, educating and training gynecologists to recognize and refer patients with psychological problems is imperative.

The limitations of this study are its small sample size (101) and non-randomized sampling, because of which the findings of the study can only be representative of the study population and cannot be extrapolated to the community at large. Longitudinal studies involving larger samples selected by systematic sampling methods would be needed to estimate the true prevalence of psychiatric morbidity and to determine the temporal association, if any between the gynecological profile and psychiatric morbidity among women attending the gynecological oncology OPD. Further studies are required to explore the relationship between female-specific cancers and psychological distress.

The findings of our study reveal a high rate of psychiatric morbidity in the gynecological oncology outpatients. Both women with benign gynecological conditions and women with gynecological cancer are at high risk of having depressive disorders and anxiety disorders compared to women from the general population. The risk for both cancer and non-cancer group seems to be more or less similar. Our findings also revealed that almost all cases of psychiatric morbidity remained undiagnosed and untreated. Hence the study emphasizes the importance of recognizing and referring the women with psychiatric morbidity by the treating clinicians in the gynecological oncology OPD clinic.
